# TRPML1 acts as a predisposing factor in lymphedema development by regulating the subcellular localization of aquaporin-3, -5

**DOI:** 10.1371/journal.pone.0310653

**Published:** 2024-12-05

**Authors:** Lijie Yang, Guanzheng Wang, Yuan Ma, Qiancheng Zhao, He Zhao, Qi Wang, Chonghua Zhong, Chunmei Zhang, Yiming Yang

**Affiliations:** 1 Department of Cell Biology and Medical Genetics, College of Basic Medical Sciences, Jilin University, Changchun, Jilin Province, China; 2 College of Basic Medicine, Changchun University of Chinese Medicine, Changchun, Jilin Province, China; 3 College of Basic Medical Sciences, Jilin University, Changchun, Jilin Province, China; The University of Texas Rio Grande Valley, UNITED STATES OF AMERICA

## Abstract

An imbalance in lymphatic fluid, whether it is caused by generation, transport, outflow, or dysfunctional vessels, can lead to lymphedema; however, the exact pathogenesis of this disease remains unclear. To explore the mechanism, we focused on the association among TRPML1, aquaporin-3 (AQP3), and aquaporin-5 (AQP5) in human lymphatic endothelial cells (HLECs). We explored the role of TRPML1 in altering the permeability of HLECs in lymphedema. Meanwhile, we constructed a disease model using gene-knockout mice to observe the effect of TRPML1 on inflammation and fibrosis in lymphedema sites. Our results indicate that TRPML1 not only regulates the localization of AQP3, -5 to the cell membrane but also increases HLEC permeability, disrupts lymphatic fluid transport, and mediates the development of chronic inflammation at the site of lymphedema. Our study suggests that TRPML1 is a precipitating factor in lymphedema. Our findings improve the understanding of TRPML1 and aquaporins in secondary lymphedema, providing valuable insights for future research.

## Introduction

Lymphedema is characterized by the accumulation of extracellular fluid in tissues due to damage or dysfunction of the lymphatic system, resulting in progressive swelling and chronic tissue inflammation. Lymphedema can be classified as primary or secondary. Secondary lymphedema is more prevalent and usually occurs after a variety of structural and functional insults to the lymphatic vasculature, including acute and chronic infections, trauma, and chronic venous hypertension [1]. These insults lead to fluid retention and accumulation in the interstitial space, resulting in local swelling and inflammation [2]. Studies have shown that lymphatic leakage and changes in lymphatic fluid transport and outflow play important roles in the development of lymphedema; excessive lymph leakage into tissues has a far-reaching impact on the disease [3, 4]. Furthermore, studies in the past decade have shown that lymphatic injury triggers a chronic inflammatory response [5, 6]. Lymphedema tissues exhibit infiltration by various inflammatory cells. For example, an increase in the number of macrophages can cause a mixed inflammatory response and tissue fibrosis, aggravating lymphatic reflux disorder [7].

Fluid metabolism plays a prominent role in the pathogenesis of lymphedema [8]. Triacca et al revealed the importance of transcellular transport mechanisms in maintaining lymphatic homeostasis and showed that human lymphatic endothelial cells (HLECs) regulate substance transport between tissues and lymph via a transcellular mechanism [9]. This suggests that water transport through the HLEC cell membrane plays a role in lymphedema. Aquaporins (AQPs) are a family of proteins that regulate water transport inside and outside cells. Recently, 13 subtypes of AQPs widely distributed in human tissues and organs have been identified. Given that water molecules are rapidly transported through membrane protein channels, AQPs are crucial in maintaining cell permeability. They are also essential for the maintenance of skin tissue, reabsorption of renal tubules, secretion of gastric acid, and inflammation [10]. An imbalance in water–electrolyte homeostasis maintained by AQPs can lead to edema. Studies have shown that hyponatremia with elevated AQP2 levels may be accompanied by edema, which is involved in abnormal water retention and ascites formation during liver cirrhosis [11]. In the central nervous system, AQP1 and AQP4 have long been associated with the pathophysiology of cerebral edema [12–15], and AQP4 contributes to the formation and regression of edema after spinal cord injury [16]. Although an increasing number of studies on AQPs and edema-related diseases have been published, to the best of our knowledge, none has clarified the role of AQPs in the complex lymphedema process, including the AQP subtypes expressed in HLECs.

Cell permeability has been considered a possible mechanism for lymphedema; studies have found a dynamic relocalization mechanism of AQPs within cells, which is a crucial process affecting cell permeability [17]. Salman et al demonstrated that changes in AQP4 localization from intracellular vesicles to the plasma membrane may affect its function, ultimately affecting cell permeability. An important feature of AQP relocalization is that AQP is transported through vesicles and fuses with the plasma membrane via exocytosis [18, 19]. The transient receptor potential (TRP) ion channel protein mucolipin-1 (TRPML1) has an important role in vesicular transport and exocytosis as it is located in the endosomal/lysosomal membrane and is a major lysosomal calcium release channel that regulates intracellular calcium concentration and membrane trafficking [20]. A recent study showed that TRPML1 induces AQP2 apical accumulation and depolymerizes the actin cytoskeleton, significantly increasing cell permeability and boosting water flux in response to hypotonic stimuli [21]. This provided inspiration for our research, leading us to investigate the water permeability of HLECs in our exploration of the possible mechanisms of lymphedema.

Beyond tissue edema, lymphedema involves chronic inflammation. Macrophages are major contributors to chronic pathological inflammation, and the activation of CD86+ macrophages appears to initiate inflammation in tissues. Macrophages produce large amounts of inflammatory cytokines in response to danger signals [22, 23], which induces irreversible damage, such as tissue fibrosis [24]. However, there has been limited research on molecular interventions involved in lymphedema, and existing clinical interventions are limited by their long duration [25]. In the present study, we aimed to explore TRPML1 function in lymphedema and further elucidate the effects of TRPML1 on the plasma membrane localization of AQP3 and AQP5, as well as its effect on alterations of water permeability in HLECs and the subsequent contribution to the process of lymphedema and associated inflammation. This may provide a direction for identifying new therapeutic targets, which is a crucial step in the development of new strategies to prevent and treat lymphedema and its associated inflammation.

## Materials and methods

### Reagents and antibodies

We purchased the following: endothelial cell medium (1001, ScienCell Research Laboratories, fetal bovine serum (0025, ScienCell Research Laboratories), endothelial cell growth supplement (1052, ScienCell Research Laboratories), Dulbecco’s modified Eagle’s medium (DMEM, E600003, Sangon Biotech), penicillin and streptomycin (0503, ScienCell Research Laboratories), Lipofectamine^TM^ 3000 (L3000075, ThermoFisher Scientific), opti-MEM (31985070, ThermoFisher Scientific), TRPML1 agonist (ML-SA1, 4746, Tocris Bioscience), TRPML1 inhibitor (ML-SI1, GW-405833, Enzo Life Sciences), bafilomycin A1 (BafA1, SC-201550, Santa Cruz Biotechnology), and Patent Blue V sodium salt (21605, Millipore-Sigma). We obtained anti-AQP3 antibody (ab125219, Abcam, Cambridge, MA, USA), anti-AQP5 antibody (20334-1-AP, Proteintech), GAPDH mouse mAb (AC002, ABclonal Biotechnology), anti-CD86 antibody (14-0862-81, ThermoFisher Scientific), anti-rat IgG SABC Kit (SA1055, Boster Biotechnology), goat anti-mouse IgG (31430, ThermoFisher Scientific), goat anti-rabbit IgG (31460, ThermoFisher Scientific), Alexa Fluor™ 488 goat anti-Rabbit IgG (H+L) (A11008, ThermoFisher Scientific), Calcein AM (C2012, Beyotime Biotechnology), Alexa fluor™ 488 phalloidin (A12379, ThermoFisher Scientific), mouse IL-1β Enzyme-linked immunosorbent assay (ELISA) kit (KE10003, Proteintech), and mouse IL-6 ELISA kit (KE10007, Proteintech). We also obtained plasmids pLV3-CMV-Aqp3-3×FLAG-GFP-Puro (P56413, Miaolingbio), pCMV-EGFP-Aqp5-Neo (P47445, Miaolingbio), membrane and cytosol protein extraction kit (P0033, Beyotime Biotechnology), and Na^+^/K^+^ ATPase Rabbit mAb (A11683, ABclonal Biotechnology).

### Cell culture

We obtained HLECs from ScienceCell and cultured them in an endothelial cell medium containing 10% endothelial cell growth supplement and 1% penicillin/streptomycin in a mixture incubated at 37°C in a 5% CO_2_ incubator. We obtained COS-1 cells from OTwo Biotech and cultured them in DMEM containing 10% fetal bovine serum and 1% penicillin and streptomycin in a mixture incubated at 37°C in a 5% CO_2_ incubator.

### Mouse tail lymphedema model

TRPML1^−/−^ (TRPML1 KO, C57BL/6J) mice were obtained from GemPharmatech Co., Ltd (T015392). Five mice per cage were housed in the animal center of the College of Basic Medical Sciences, Jilin University, with free access to food and water and with a controlled room temperature (RT, 22 ± 4°C), 50–60% humidity, under an automatically controlled light cycle (light on 06:00–18:00 h).

Male mice were used at 6 weeks of age and underwent tail lymphedema induction. Briefly, the mice were continuously anesthetized using an inhalation anesthesia machine. The anesthesia included pure oxygen, the isoflurane was volatilized through the oxygen flow, and the anesthesia concentration was 2%. Patent Blue solution (1 mg/mL) was injected into the tail tip to visualize the lymphatic vessels, and operated on in the following groups: SHAM: wild-type (WT) littermate mice sham-operated group, involving a circular incision of the epidermal layer only, without epidermal removal at 1.5 cm from the base of the tail (n = 6); WT: WT littermate mouse-operated group, with the excision of a 2-mm-wide circular full-length layer of tail skin at approximately 1.5 cm from the base of the tail to damage the lymphatic vessels (n = 6); TRPML1^−/−^: TRPML1-knockout mouse surgical group with the same treatment as the WT group (n = 6). Three mice per cage from the same group were housed after operation. The area around the surgical incision was wrapped with a transparent dressing (1626WNS, 3M^TM^ Tegaderm^TM^, Japan) to prevent infection, and the dressing was removed after 24 h [26]. The tail diameter of the mice with lymphedema was measured using a Vernier caliper every three days until day 27. The tail was soaked in hot water at 60°C for 1 min and dried. Then, the tail tip was excised for approximately 1 cm, and blood from the tail vein was collected. All mice were euthanized using CO_2_ according to the “standard procedure for carbon dioxide Euthanasia of Laboratory Animals”. One segment of the edematous part of the tail was extracted for RNA, and the other segment was fixed in 4% paraformaldehyde for 48 h, decalcified with 10% EDTA at room temperature for 15 days, embedded in paraffin, and stained with hematoxylin and eosin or prepared for immunohistochemical analysis. The animal study protocol was approved by the Ethics Committee of Collage of Basic Medical Sciences, Jilin University (AP# 2023–530).

### qPCR assays

RNA from HLECs was extracted, reverse-transcribed, and subjected to qPCR assays. Data were analyzed using the ΔΔCT relative quantification method. The primer sequences are listed in Table 1. The qPCR products were analyzed using 1.5% agarose gel electrophoresis.

**Table 1:** Primer sequences for HLECs qPCR.

Gene	Sequence 5′-3′
**GAPDH**	Forward Primer: GGTTGTCTCCTGCGACTTCA
	Reverse Primer: TGGTCCAGGGTTTCTTACTCC
**AQP0**	Forward Primer: TTGCCACATACGACGAGAGG
	Reverse Primer: CCTGCACCAGTATAATACATCCC
**AQP1**	Forward Primer: CCTGGCTGTACTCATCTACGACTTC
	Reverse Primer: CCGCTGGTCCACACCTTCAC
**AQP2**	Forward Primer: ACCAGGCAATACCCATCCATCAC
	Reverse Primer: GAGGCAGACAGAGAAGGAACAGAG
**AQP3**	Forward Primer: CTGTCACTCTGGGCATCCTCATC
	Reverse Primer: TCACGAGCCAGGAAGCACATG
**AQP4**	Forward Primer: GGGTCTATCGCCTTGTGGATGG
	Reverse Primer: GTGTATCTGTCAGCAGTGGTCTCC
**AQP5**	Forward Primer: GCTGGCATCCTCTACGGTGTG
	Reverse Primer: CCCTGCGTTGTGTTGTTGTTGAG
**AQP6**	Forward Primer: TTCTTTGGCGTGGGCTCAGTC
	Reverse Primer: GGCGGTGACCAGGTTGAAGG
**AQP7**	Forward Primer: CCAAGGCGGAGGCTGAGAATC
	Reverse Primer: ATCTTTGCTATCACGGACCAGGAG
**AQP8**	Forward Primer: TGACTGCTGAGGAGGCTCTAGG
	Reverse Primer: CGCTCGTGTTGTGGTGTTTGC
**AQP9**	Forward Primer: GCTCCGTATCTATCTCTGGCGAAC
	Reverse Primer: CCGATGGCAATGGGCTCTAGG
**AQP10**	Forward Primer: AACTGCGGGATTCCACTCAACC
	Reverse Primer: CACCCACCACCAGCCATTACC
**AQP11**	Forward Primer: TGTCGGTGGTGCTGCTCATG
	Reverse Primer: GGCTAGAAACTCCAGGACGAAGG
**AQP12A**	Forward Primer: CTGCTCGGGACACACCTTACTG
	Reverse Primer: GCAGCACAGCCAGGACCATC
**AQP12B**	Forward Primer: CTGCTCGGGACACACCTTACTG
	Reverse Primer: GCAGCACAGCCAGGACCATC

Mouse tail edema parts were fragmented, added to TRIzol reagent and ceramic beads (3 mm), and subjected to homogenization in a tissue homogenizer at −20°C, 70 Hz, and 180 s, and this was repeated four times. Centrifugation was performed to extract RNA from the supernatant, which was reverse-transcribed to DNA. We then performed qPCR on whole-tissue extracts from lymphedema mouse tails using AQP3, AQP5, IL-1β, IL-6, COL1, vimentin, α-SMA, and TGF-β1 primers. Data were analyzed using the ΔΔCT relative quantification method. The primer sequences are listed in Table 2.

**Table 2:** Primer sequences for qPCR of lymphedema tissues.

Gene	Sequence 5′-3′
**GAPDH**	Forward Primer: AGGTCGGTGTGAACGGATTTG
	Reverse Primer: GGGGTCGTTGATGGCAACA
**AQP3**	Forward Primer: CCTTGGCATCTTGGTGGCT
	Reverse Primer: AGGAAGCACATTGCGAAGGT
**AQP5**	Forward Primer: TCTTGTGGGGATCTACTTCACC
	Reverse Primer: TGAGAGGGGCTGAACCGAT
**IL-1β**	Forward Primer: GCAACTGTTCCTGAACTCAACT
	Reverse Primer: ATCTTTTGGGGTCCGTCAACT
**IL-6**	Forward Primer: TAGTCCTTCCTACCCCAATTTCC
	Reverse Primer: TTGGTCCTTAGCCACTCCTTC
**COL1**	Forward Primer: GCTCCTCTTAGGGGCCACT
	Reverse Primer: CCACGTCTCACCATTGGGG
**Vimentin**	Forward Primer: CGTCCACACGCACCTACAG
	Reverse Primer: GGGGGATGAGGAATAGAGGCT
**α-SMA**	Forward Primer: GTCCCAGACATCAGGGAGTAA
	Reverse Primer: TCGGATACTTCAGCGTCAGGA
**TGF-β1**	Forward Primer: CTCCCGTGGCTTCTAGTGC
	Reverse Primer: GCCTTAGTTTGGACAGGATCTG

### Western blotting assays

HLECs were cultured in six-well plates. At 80% cell confluence, each group of cells, excluding the negative control (NC) group, received the following treatments: ML-SA1 (30 μM), ML-SI1 (25 μM), ML-SI1 (25 μM) and ML-SA1 (30 μM) co-incubation, and BafA1 (0.1 μM) for 24 h. Subsequently, total HLEC cellular protein was extracted (The homogenate was then centrifuged at 12,000 g for 30 min and the supernatants were collected. Extraction of surface and cytoplasmic proteins was conducted using the surface and cytoplasmic protein reagent kit according to the manufacturer’s instructions.), and 20 μg of protein was added to each polyacrylamide gel well. After proteins were separated through SDS-PAGE, they were transferred to a PVDF membrane. The PVDF membrane was then blocked with 5% skim milk for 2 h and incubated with AQP3, AQP5, and GAPDH antibodies overnight (4°C), followed by incubation with an HRP antibody for 2 h (RT). All primary antibodies were used at a 1:2000 dilution in diluent (GF1600-01, Genefist), and secondary antibodies were diluted at 1:5000 in TBST. Finally, ECL was used to visualize the PVDF membranes.

### Immunofluorescence and confocal microscopy

The HLECs, which were cultured on coverslips in 24-well plates, and cells at 50% confluence received the following treatments: ML-SA1 (30 μM) for 30 min, ML-SI1 (25 μM) for 30 min either alone or followed by co-incubation with ML-SA1 (30 μM), and BafA1 (0.1 μM) for 30 min. The cell monolayer was then fixed with 4% paraformaldehyde for 15 min (RT). Cells were blocked with 5% BSA at 37°C for 40 min. The primary antibody was incubated overnight (4°C), the secondary antibody was incubated at 37°C for 1 h (Dark), and the coverslips were mounted with antifade reagent (S210, Solarbio, Beijing, China). Images were captured using an Olympus confocal microscope (FV3000).

The Cos-1 cells were cultured in 24-well plates, and cells at 50% confluence were subjected to transient overexpression of AQP3 or AQP5. Then, 300 ng pLV3-CMV-Aqp3-3×FLAG-GFP or 300 ng pCMV-EGFP-Aqp5 plasmids with 0.3 μL P3000 reagent and 0.6 μL Lipofectamine^TM^ 3000 reagent was diluted in 25 μL opti-MEM and incubated for 15 min. The transfection complexes were added to the cells and incubated for 6 h before adding fresh medium. After transfection for 24 h, cells received the following treatments: ML-SA1 (30 μM) for 30 min, ML-SI1 (25 μM) for 30 min either alone or followed by co-incubation with ML-SA1 (30 μM), and BafA1 (0.1 μM) for 30 min. The cell monolayer was then fixed with ice-cold methanol for 15 min (4°C). The coverslips were mounted with antifade reagent and images were captured using an Olympus confocal microscope (FV3000).

### Calcein-AM (Cal-AM) staining assays

HLECs were cultured in 24-well plates, and at 90% cell confluence, they received the treatments described in the section “Immunofluorescence and confocal microscopy”. Subsequently, the cells were incubated with Cal-AM (2 μM) for 15 min at 37°C and washed three times with PBS. Fluorescence values were then detected using a microplate reader at an excitation light of 494 nm and emission light of 514 nm.

HLECs cultured in 12-well plates were stained with Cal-AM at 50% cell confluence, washed four times with PBS containing calcium, and subjected to the treatments described in the section “Immunofluorescence and confocal microscopy”. The cells were then placed under a fluorescence microscope connected to a SharpCap 3.0 system. A hypotonic stimulus was applied almost simultaneously with the start of image recording, capturing images every 1 s for 7.5 min. The resulting graphs were merged into videos using Adobe Premiere Pro CC 2017.

### Immunohistochemistry assay

The mouse tail tissues were dewaxed in xylene and subjected to antigen retrieval. Between all steps, the tissue sections were washed using PBS (pH 7.2–7.6). Slides were incubated with 5% BSA for 60 min, and then incubated with anti-CD86 antibody overnight at 4°C. Then, tissue sections were incubated with anti-rat IgG for 2 h at 37°C, followed by streptavidin-biotin complex for 20 min at 37°C. Color reactions from applying 5-bromo-4-chloro-3-indolylphosphate and nitro blue tetrazolium were observed. Tissue sections were air-dried and sealed with coverslips using the water-soluble mounting medium. Finally, the slices were observed under the microscope. CD86+ cells were visible as brown staining. The immunostaining results were analyzed based on the average number of positive cells using ImageJ [27].

### Enzyme-linked immunosorbent assay (ELISA)

Blood from the tail veins of mice was centrifuged at 3000 rpm and 4°C for 20 min. The serum was collected, and ELISA was performed according to the kit’s instructions. Briefly, the sample was added to the well plate and incubated for 2 h at 37°C. The ELISA plate was washed three times and incubated in detection antibody for 1 h at 37°C. Then, the plate was washed and treated with HRP-labeled streptavidin and incubated for 40 min at 37°C. After three washes, the plate was developed using TMB One Solution substrate, and the reaction was stopped with stopping solution. The absorbance was measured at 450 nm using a microplate reader (Synergy H1).

### Statistical analysis

All data were obtained from three experiments and are presented as the mean ± standard deviation. GraphPad Prism (version 9.0) was used to perform statistical analyses. Statistical analysis was carried out using t-test to assess the significance of outcomes between groups. *p* < 0.05 represented a significant difference between groups.

## Results

### TRPML1 is positively related to mouse lymphedema

**Fig 1:**
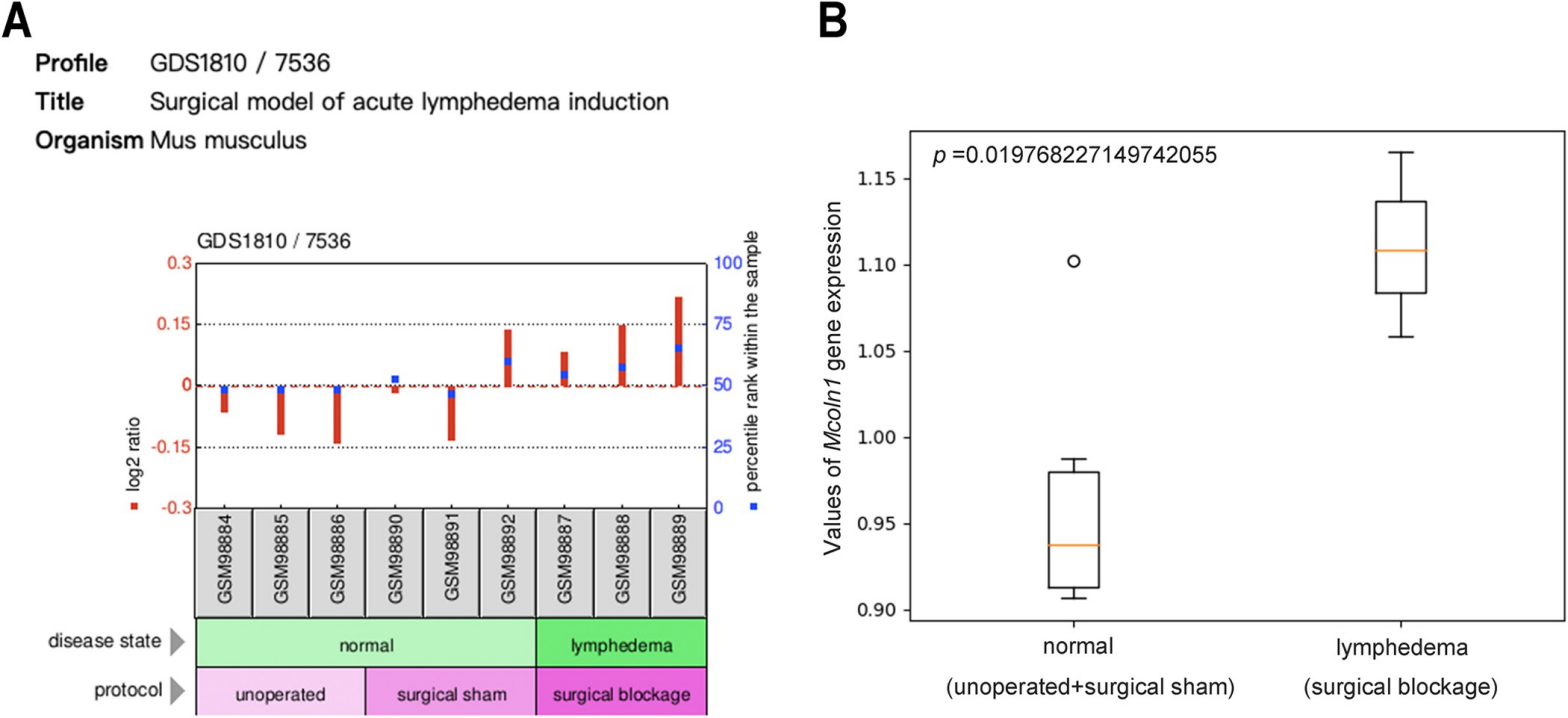
Gene expression of *Mcoln1* in lymphedema tissue and normal tissue. **(A)** *Mcoln1* expression profiling by microarrays from GEO database (GSE4333). Three sets of microarrays with three replicates each for a total of 9 arrays were performed. The three conditions were normal tail skin (no intervention), lymphedema tail skin (due to surgical lymphatic vessel blockage), and surgical sham control tail skin (surgical incision with no lymphatic vessel blockage). **(B)** Data visualization was performed using Python to perform t-test. *Mcoln1* expression is higher in the lymphedema group than in the normal group (*p* < 0.05).

To explore whether TRPML1 is associated with lymphedema, we downloaded microarray datasets (GSE4333) from the Gene Expression Omnibus (GEO) database. As shown in Fig 1(A) and 1(B), the expression level of the mucolipin TRP cation channel 1 *(Mcoln1)* gene, which encodes TRPML1, was higher in lymphedema tail skin than in normal tail skin. The high expression of TRPML1 in lymphedema tissue suggested that TRPML1 might be implicated in lymphedema progression.

### TRPML1^−/−^ mice showed weaker symptoms of lymphedema

**Fig 2:**
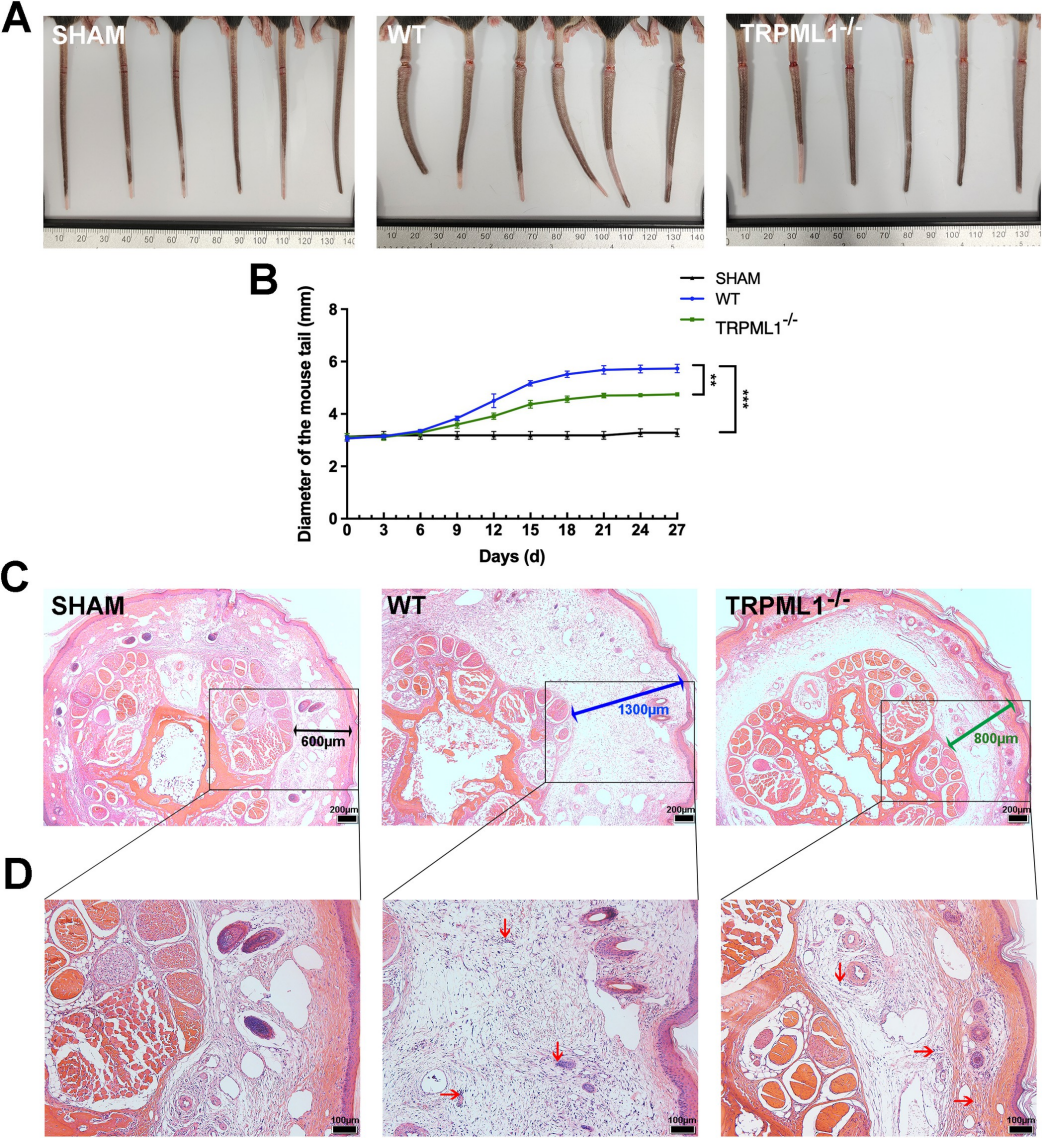
Pathological changes in the tail of lymphedema mouse model. SHAM: Sham operation group; WT: Wild-type mouse model group; TRPML1^−/−^: TRPML1 gene-knockout mouse model group. **(A)** Comparison of actual tail conditions of lymphedema mice in each group on day 27. The tails of mice in the SHAM group showed no tissue edema, and the tails showed a natural shape. The tails of mice in the WT group showed unnatural bending and severe tissue edema. The tails of TRPML1^−/−^ mice were able to naturally straighten, and the symptoms of tissue edema were significantly weaker than those of WT mice. **(B)** Diameter changes in the cross-section of mouse tail edema. From days 0 to 27, the tail diameters of mice in the SHAM group did not change, while those of mice in the WT and TRPML1^−/−^ groups significantly increased on day 9, peaked on day 21, and then plateaued. The curve of the WT group rose (the difference between the WT group and SHAM group was significant, ***p < 0.001), while the curve of the TRPML1^−/−^ group showed a more gradual increase, with the degree of whole tail edema being lower (the TRPML1^−/−^ and WT groups were significantly different, **p < 0.01). **(C)** Hematoxylin and eosin (HE) staining results of the cross-section of lymphedema sites in mice. Images were viewed with an Olympus BX53 microscope with an Olympus UPlanFLN 4×/0.13 objective. The average interstitial thickness of the SHAM group was ~600 μm (black arrow). The average interstitial thickness in the WT group significantly increased to ~1300 μm (blue arrow). The average interstitial thickness of the TRPML1^−/−^ group was smaller than that of the WT group, ~800 μm (green arrow). Scale bar = 200 μm. **(D)** HE staining results of the cross-sections of lymphedema sites in mice. Images were viewed with an Olympus BX53 microscope with an Olympus UPlanFLN 10×/0.30 objective. In the SHAM group, the cross-sectional structure of the tail was complete, compact, and regularly arranged, and there was almost no inflammatory cell infiltration. In the WT group, the staining structure was incomplete, the interstitial tissue was obviously expanded, the collagen fiber was seriously broken, and many inflammatory cells were infiltrated (red arrow). In the TRPML1^−/−^ group, the main tissue structure was undamaged, and the degrees of tissue interstitial expansion and inflammatory cell infiltration were slight. Scale bar = 100 μm.

To further explore the correlation between TRPML1 and lymphedema, we constructed a lymphedema tail model using TRPML1 gene knockout (TRPML1^−/−^) and WT mice. The mouse genotypes identified through PCR are shown in S1 Fig. As shown in Fig 2A, the WT mice exhibited significant tail edema compared with those in the SHAM group, and the internal pressure from swelling caused the tail to bend to one side. However, the degree of tail edema was relatively low in the TRPML1^−/−^ mice. Fig 2B illustrates the onset of tail edema in mice on day 6 after lymphatic ligation, with observable differences in tail diameter on day 9. The difference in tail diameter among the groups was most significant from day 18, remaining consistent from day 21. As shown in Fig 2C, hematoxylin and eosin staining of mouse lymphedema tissues showed that compared with that in the SHAM group, the subcutaneous tissue gap of WT mice was significantly widened, the tissue was excessively loose, the collagen fibers in the interstitium were swollen, and a part of the structures was disintegrated. The edema symptoms of TRPML1^−/−^ mice were significantly alleviated, the diameter of the tail was slightly increased, the main internal structures of the tail tissue remained clear and complete, and the interstitial space was significantly narrowed. The WT mice had severe lymphedema symptoms, while the TRPML1^−/−^ mice had significantly weaker symptoms. These results indicate that TRPML1 plays a significant role in the development of lymphedema.

### AQP3, -5 are the main subtypes of AQPs highly expressed in HLECs

**Fig 3:**
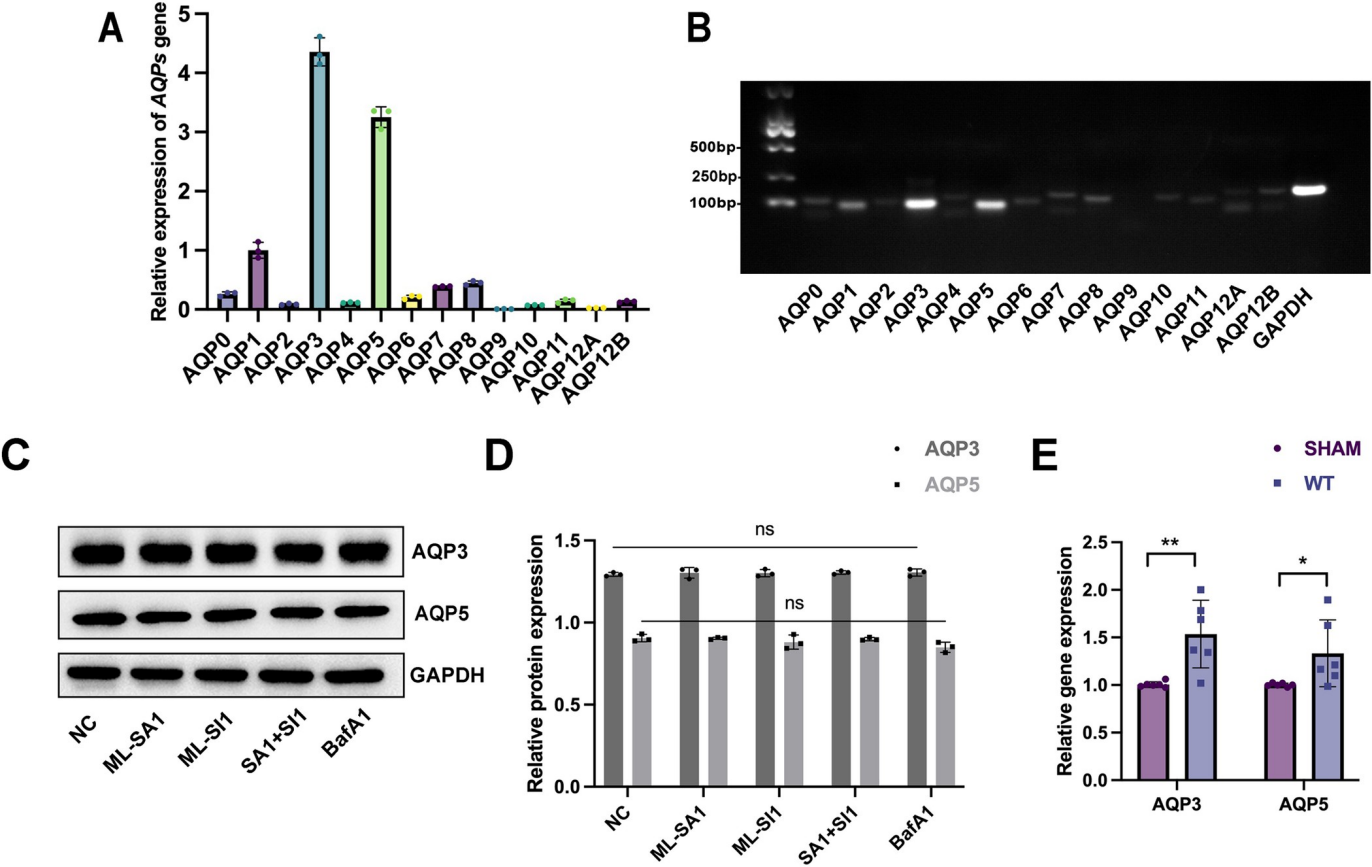
Gene and protein expression of AQPs in HLECs. **(A)** Gene expression of *AQP1-12B* in HLECs was assessed using qPCR with *GAPDH* as the housekeeping gene. *AQP3* gene expression was the highest, followed by *AQP5* expression. **(B)** Gene expression of *AQP1-12B* in HLECs was analyzed through agarose gel electrophoresis. The PCR products were approximately 100 bp, and the results were consistent with those of qPCR. **(C)** The effect of TRPML1 on the expression of AQP3, -5 protein was analyzed through western blot using GAPDH as a control. **(D)** Statistical analysis of western blot strips is shown. HLECs were treated for 24 h according to the following groups: NC: negative control; ML-SA1: 30 μM; ML-SI1: 25 μM; ML-SA1+ ML-SI1: 25 μM ML-SI1 and 30 μM ML-SA1 co-incubated; BafA1: 0.1 μM **(E)** Gene expression of *AQP3*, *-5* in mouse lymphedema tissue was assessed using qPCR. Compared with that in the SHAM group, *AQP3*, *-5* gene expression in lymphedema tissue of WT mice showed a tendency to increase.

Changes in lymphatic endothelial cell fluid permeability were considered after the phenomenon of tissue fluid retention caused by lymphatic vessel injury was observed in the mouse lymphedema model. To screen the most likely AQP subtypes in lymphedema, we designed 13 PCR primers targeting known AQR subtypes and performed qPCR and agarose gel electrophoresis assays on HLECs. Fig 3(A) and 3(B) shows that the highest relative expression of AQP subtypes in HLECs was that of AQP3 and AQP5. Consequently, we focused on the effect of TRPML1 on AQP3, -5 in subsequent experiments. We treated HLECs with ML-SA1 (a TRPML1 agonist), ML-SI1 (a TRPML1 inhibitor), and BafA1 (an inhibitor of vacuolar H+-ATPase) for 24 h, and we used western blot to detect the protein expression of AQP3, -5. The results in Fig 3C and 3D show that the overall protein expression of AQP3, -5 did not significantly change when TRPML1 was activated or inhibited or when lysosomal function was inhibited. This implies that TRPML1 does not directly influence lymphedema through modulating AQP3, -5 protein expression levels.

Following induction of the lymphedema model, the lymphedema tissue of mice was retained, and the gene expression changes of *AQP3*, *-5* were detected by qPCR using the SHAM group as the control group. The results in Fig 3E validated the potential involvement of *AQP3*, *-5* in lymphedema in mice.

### TRPML1 promoted AQP3, -5 accumulation on the membrane and depolymerized the actin cytoskeleton

**Fig 4:**
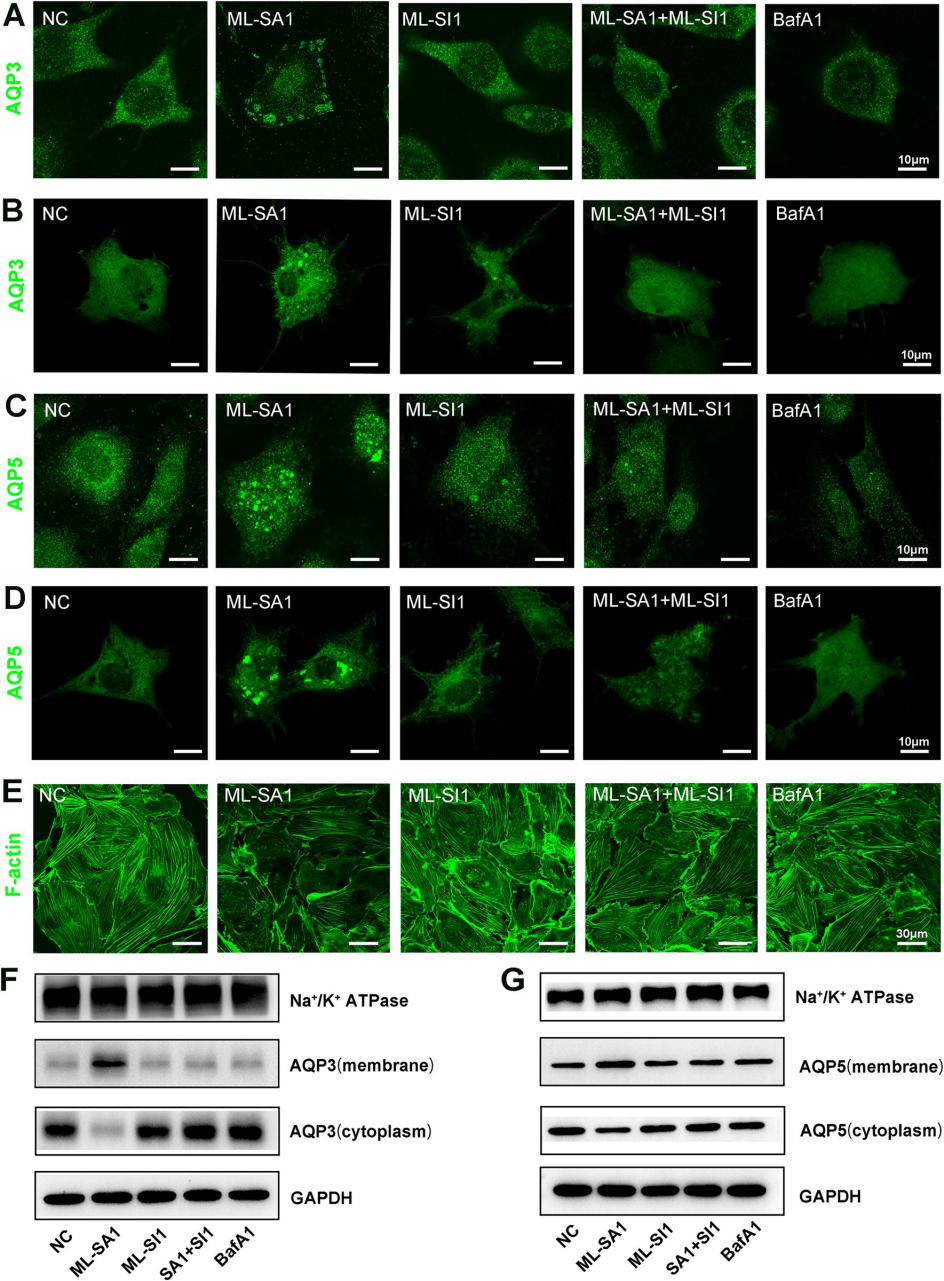
Localization changes in AQP3, -5 in HLECs and actin skeleton staining. HLECs and transfected Cos-1 cells were treated in the following groups: NC: negative control; ML-SA1: 30 μM for 30 min; ML-SI1: 25 μM for 30 min; ML-SA1+ ML-SI1: 25 μM ML-SI1 and ML-SA1 co-incubated for 30 min; BafA1: 0.1 μM for 30 min. **(A)** Subcellular localization changes in AQP3 in HLECs. In the NC and BafA1 group, AQP3 fluorescence was mostly distributed in the cytoplasmic region. The AQP3 fluorescence of the ML-SA1 group was distributed diffusively throughout the cell and accumulated on the surface of the cell membrane. ML-SI1 alone or with ML-SA1 did not change the fluorescence range of AQP3. Scale bar = 10 μm. **(B)** Cos-1 cells were transfected with AQP3-GFP plasmids, and the membrane orientation of AQP3 under each treatment was observed. Only the ML-SA1-treated cell showed obvious high-density green fluorescent patches on the membrane surface. Scale bar = 10 μm. **(C)** Subcellular localization changes in AQP5 in HLECs. In the NC and BafA1 group, AQP5 fluorescence was slight and diffused around the cell. According to the fluorescence distribution of the ML-SA1 group, AQP5 accumulated to the cell membrane. ML-SI1 alone or with ML-SA1 did not change the fluorescence range of AQP5. Scale bar = 10 μm. **(D)** Membrane orientation changes in the overexpression of AQP5 in Cos-1 cells. Only the ML-SA1-treated cell showed obvious high-density green fluorescent patches on the membrane surface. Scale bar = 10 μm. **(E)** Phalloidin staining of the cytoskeleton in HLECs. In the NC group, the fluorescence of phallus cyclic peptide was bright, and the whole cytoskeleton was clear. In the ML-SA1 group, the fluorescence of phallus cyclic peptide was dim, and the cytoskeleton disappeared or began to break. ML-SI1 alone or with ML-SA1 did not cause changes in the cytoskeleton; BafA1 also did not show any significant effect on the cytoskeleton. Scale bar = 30 μm. **(F)** The membrane portions of AQP3 following activation of TRPML1 were measured by western blot. **(G)** The membrane portions of AQP5 following activation of TRPML1 were measured by western blot. HLEC membrane proteins and plasma proteins were extracted separately to analyze the distribution of AQP3, -5. After ML-SA1 stimulation, both AQP3, -5 were more detected in the components of membrane proteins, and AQP3, -5 were more present in the form of polymers on the cell membrane, while relatively less AQP3, -5 was detected in the cytoplasm.

Although our initial experimental results showed that TRPML1 did not directly affect the overall protein expression of AQPs, the literature indicates that the function of AQPs must be localized to the cell membrane. Thus, we conducted immunofluorescence experiments using confocal microscopy to assess the subcellular localization of AQP3, -5 after a series of treatments. As shown in Fig 4(A), 4(C) and 4(E), the activation of TRPML1 by ML-SA1 causes membrane accumulation of AQP3, -5 and significant depolymerization of the actin cytoskeleton, which is likely to be associated with increased cell water permeability. Notably, ML-SI1 alone did not exhibit inhibitory effects but completely prevented ML-SA1-induced AQP3, -5 plasma membrane accumulation and cytoskeletal depolymerization. The lysosomal function inhibitor BafA1 tended to have no effect on membrane localization of AQP3 and AQP5 without affecting the cytoskeleton.

To further clarify the influence of TRPML1 on the membrane surface orientation of AQP3, -5, we overexpressed AQP3 or AQP5 in Cos-1 cells, as shown in Fig 4(B) and 4(D). The results clearly showed that after the activation of TRPML1 by ML-SA1, highly fluorescent patches of AQP3 and AQP5 appeared on the surface of Cos-1 cells, while the cells in other groups did not show obvious membrane localization compared with those in the blank group.

To further confirm the recruitment of AQP3 and AQP5 to the cell membrane, we extracted membrane portion proteins of HLECs. As shown as Fig 4F and 4G, western blot results showed that AQP3 and AQP5 were the most expressed in the membrane under the stimulation of ML-SA1. These results were consistent with immunofluorescence results in HLECs.

### TRPML1 increased water permeability in HLECs

**Fig 5:**
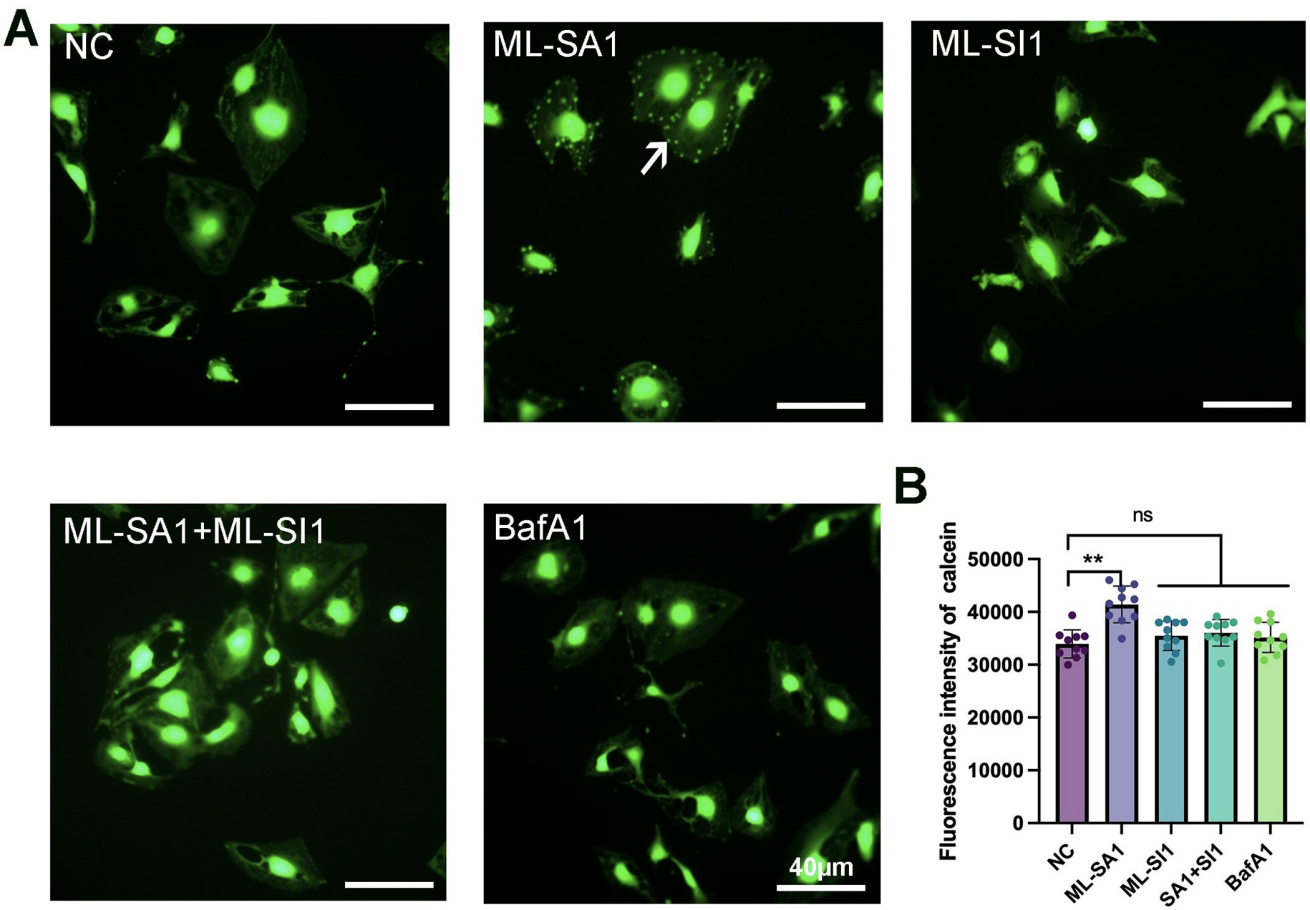
Fluorescence changes in HLECs stained by Cal-AM. HLECs were treated in the following groups: NC: negative control; ML-SA1: 30 μM for 30 min; ML-SI1: 25 μM for 30 min; ML-SA1+ ML-SI1: 25 μM ML-SI1 and ML-SA1 co-incubated for 30 min; BafA1: 0.1 μM for 30 min. **(A)** The fluorescence of HLECs was recorded at the 26th second. Only the ML-SA1-treated cells showed a transient green fluorescence signal around the membrane after hypotonic stimulation (white arrow), while the other groups of cells did not show any change in fluorescence signal after hypotonic stimulation. Scale bar = 40 μm. **(B)** The fluorescence intensity of HLECs was detected using a microplate reader. Compared with that of the NC group, the fluorescence intensity of the ML-SA1 group was enhanced, and the difference was significant (**p < 0.01). There was no significant difference in fluorescence intensity in the other three groups compared with that in the NC group (*p* > 0.05).

Quantifying biofilm permeability using Cal-AM is a sensitive and reliable technique for assessing the transmembrane permeability of cells [28, 29]. In the present study, HLECs were pre-stained with Cal-AM and treated as indicated. As shown in Fig 5A, fluorescence rapidly appeared in the HLEC membrane area of cells treated with ML-SA1 only after exposure to hypotonic stimulation. Subsequently, the overall fluorescence brightness gradually decayed and darkened with time, as in the other groups. The fluorescent changes are shown in S1–S5 Videos. We speculate that the transient appearance of small, bright spots around the membrane was due to the formation of a sudden and large amount of water flux across the membrane, which was not observed in the other treatment groups.

We investigated the effect of TRPML1 on membrane permeability by incubating HLECs with Cal-AM for 15 min after hypotonicity stimulation. As shown in Fig 5B, the calcein fluorescence intensity in the ML-SA1 group was higher than that in the NC group, indicating that TRPML1 activation increased the water permeability of the HLEC membrane. ML-SI1 isolates the effects of cell permeability without inhibition but inhibits the effects of ML-SA1. However, BafA1 restricted the membrane localization of AQP3, -5; therefore, the water permeability of the cells was not enhanced. This further demonstrates that the activation of TRPML1 enhances the water permeability of HLECs by mediating the membrane aggregation of AQP3, -5, contributing to the pathological process of lymphedema.

### TRPML1 knockout attenuated lymphedema inflammation and prevented inflammatory fibrosis in mice

**Fig 6:**
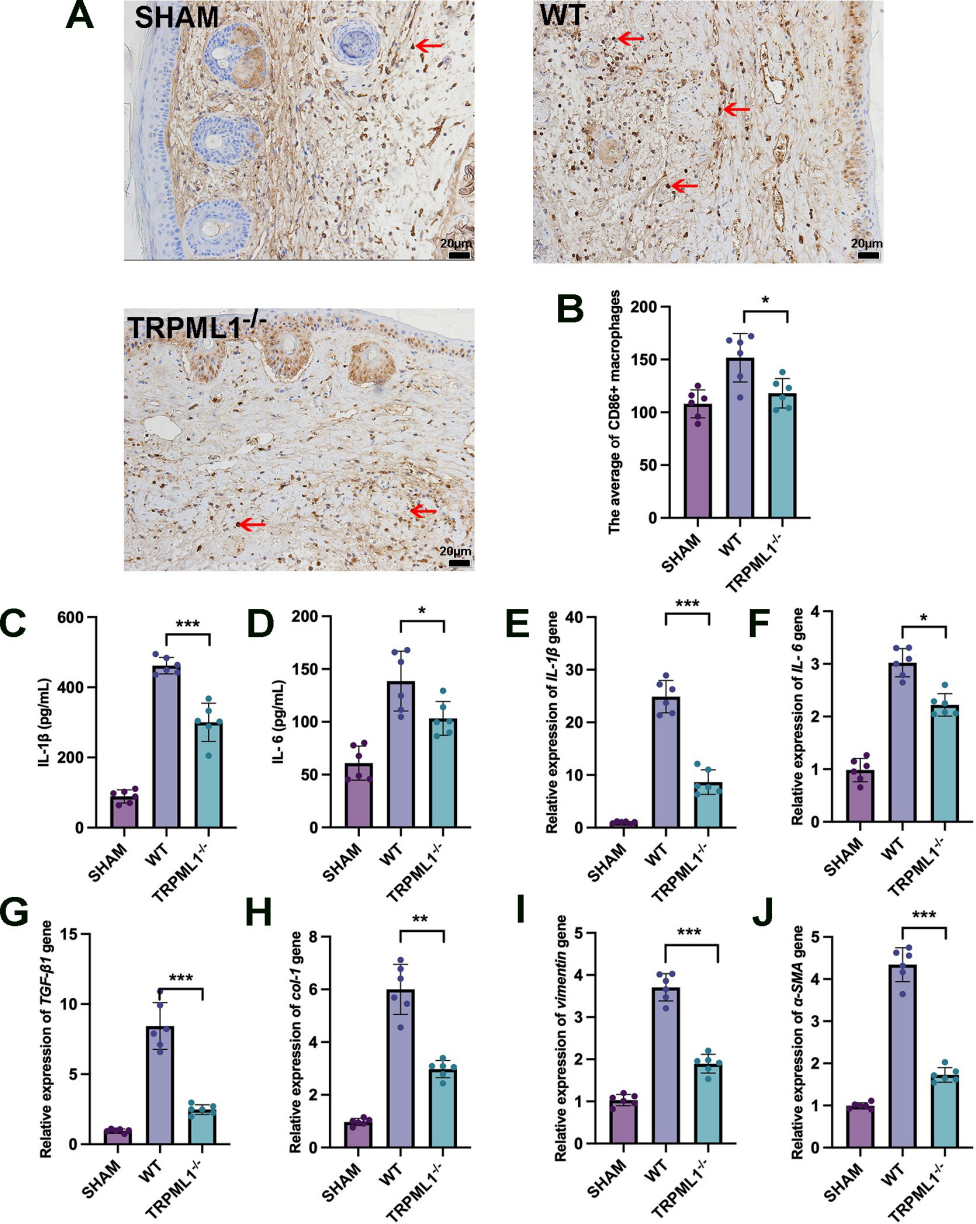
Effect of TRPML1 on inflammation in lymphedema mice. SHAM: sham operation group; WT: wild type mice model group; TRPML1^−/−^: TRPML1 gene knockout mouse model group. **(A)** Representative figure of CD86+ macrophage infiltration in the cross-sections of lymphedema sites in mice. In the SHAM group, a few CD86+ macrophages infiltrated. In the WT group, CD86+ macrophages infiltrated (red arrow). In the TRPML1^−/−^ group, the CD86+ macrophage infiltration was slight. Scale bar = 20 μm. **(B)** Statistical analysis of immunohistochemistry assay. The average of CD86+ macrophages in the WT group was higher than that in the SHAM group, while the average of macrophages in the TRPML1^−/−^ group was significantly less than that in the WT group (*p < 0.05). **(C, D)** Serum IL-1β/IL-6 secretion was detected through ELISA. The concentration of IL-1β/IL-6 in the SHAM group was normal. Compared with that in the SHAM group, the IL-1β/IL-6 concentration in the WT group significantly increased. The concentration of IL-1β/IL-6 in the TRPML1^−/−^ group was significantly lower than that in the WT group (*p < 0.05, ***p < 0.001). **(E–J)** The relative expression of IL-1β/IL-6/TGF-β1/col-1/vimentin/α-SMA genes in mouse lymphedema tissues was detected using qPCR. Compared with that of the SHAM group, the expression of IL-1β/IL-6/TGF-β1/col-1/vimentin/α-SMA genes in the WT group was significantly increased. Compared with that in the WT group, the expression of IL-1β/IL-6/TGF-β1/col-1/vimentin/α-SMA genes was significantly decreased in the TRPML1^−/−^ group (*p < 0.05, **p < 0.01, ***p < 0.001).

Although TRPML1 mediates the development of lymphedema by enhancing the water permeability of HLECs, the relationship between TRPML1 and inflammation within the edematous tissue remains unclear. The pathological characteristics of lymphedema manifest in tissue edema and, more importantly, in persistent chronic inflammation. Previous studies have shown that CD86 is specifically expressed on macrophages during chronic inflammation and is useful as a macrophage marker [30]. Therefore, we performed immunostaining of CD86 using tissue sections from the mouse tail. As shown in Fig 6(A) and 6(B), CD86+ macrophages were more infiltrated in the WT group than in the SHAM group, and TRPML1 knockout could reduce macrophage infiltration during chronic inflammation, suggesting that TRPML1 knockout may attenuate lymphedema inflammation.

To further explore the effect of TRPML1 on inflammatory infiltrate in lymphedema tissues, serum ELISA and tissue qPCR were performed in our lymphedema mouse model to detect inflammation-related indicators, including the release of the typical inflammatory factors IL-1β and IL-6, as well as the tissue fibrosis markers TGF-β1, collagen 1 (col-1), vimentin, and α-SMA [31, 32]. According to the results (Fig 6C–6J), serum inflammatory factors and tissue fibrosis levels of mice in the model group were significantly increased compared with those in the SHAM group. Conversely, serum inflammatory factor secretion in mice with lymphedema after TRPML1 knockout was effectively inhibited, and tissue inflammatory fibrosis was simultaneously inhibited.

## Discussion

The ultimate cause of lymphedema, particularly secondary lymphedema, is damaged or surgically removed lymphatic vessels, which manifests clinically as tissue swelling [33]. In the present study, we analyzed data from the GEO database and found that lymphedema is closely related to the *Mcoln1* gene and we successfully constructed a mouse model of lymphedema by disrupting the lymphatic vessels in the tail, revealing milder edema symptoms in TRPML1-knockout mice. We hypothesized that there is some regulation of lymphedema by TRPML1. Previous reports have suggested that dysfunctional lymphatic vessels cause excessive leakage of lymphatic fluid, leading to pathological accumulation of interstitial fluid in tissues [4, 34]; however, the molecular processes involved lack certain links, so it is particularly important to explore the relationship between altered lymphatic permeability and lymphedema. The regulation of osmolality and water homeostasis is dependent on AQPs, facilitating fluid accumulation and drainage. Previous studies have reported that silencing the *AQP5* gene leads to a 49.4% reduction in water permeability in human lung adenocarcinoma cells [28]. Successive reports of AQPs associated with edema have appeared in recent years [35–39]; despite this, studies related to AQPs and lymphedema are mostly absent. Furthermore, previous investigations into lymphedema have exclusively focused on regulating whole lymphatic vessel permeability by, for example, VE-cadherin, VEGFR3, and ROCK2, emphasizing cell–cell junction-dependent regulation [3, 40–42].

Our findings suggest that alterations in water permeability within cells may contribute to lymphedema development. AQPs are the major factors affecting cellular water permeability. We reviewed the data and found that subcellular trans-localization of AQPs, achieved through vesicular transport to engage and fuse with membranes, is a ubiquitous regulatory mechanism in the mammalian AQP family [43–45]. Our preliminary results showed that lymphatic endothelial cells in particular have high expression of AQP3 and AQP5, two aquaporin proteins that are more commonly discussed in disease research models, but low expression levels of other proteins. Based on these, our study used TRPML1 as an upstream target of AQP3, -5 molecules to conduct experiments. The results showed that, as an important molecule for vesicular transport [21], TRPML1 could indeed mediate the accumulation of AQP3, -5 at the plasma membrane of HLECs. This regulation led to increased cellular permeability, influencing the development of lymphedema. Our findings were supported by results from TRPML1 knockout in mice, showing a significant trend toward the attenuation of lymphedema, even with similarly damaged lymphatic vessels.

Nearly all cases of lymphedema are accompanied by inflammation and tissue fibrosis [46]. AQPs are associated with inflammatory processes [47], and the TRPML family has a background in immune regulation [48, 49]. Our results similarly indicated the involvement of TRPML1 in regulating the chronic inflammatory process in lymphedema. It is not difficult to determine the persistence of TRPML1’s effect on chronic inflammation by quantifying the level of CD86+ macrophage infiltration and tissue fibrosis in the post-inflammatory phase. Previous studies have shown that TRPML1 controls the apical fusion of AQPs on the membrane by activating the Ca^2+^/calcineurin/NFAT pathway, which leads to depolymerization of the actin cytoskeleton [21], or through the AVP/V2R/cAMP/PKA signal axis to phosphorylate AQPs [45, 50]. We believe that local Ca^2+^ release by TRPML1 is the decisive factor for AQP3, 5 transport. This is not only because TRPML1 itself plays a series of roles as a calcium channel, but also because calcium is involved in the regulation of actin dynamics; however, the specific molecular mechanism still requires further studies, and such knowledge would guide the development of molecular drugs to treat lymphedema.

This study had some limitations. First, at the initial stage of the study, we hypothesized that TRPML1 mediates the development of lymphedema using a knockout mouse model. However, clinical data support for this hypothesis was deficient because the collection of data from patients with clinical lymphedema is a lengthy process, and there is not yet a database regarding the correlation between gene expression and lymphedema incidence. We hope that in the future, a lymphedema clinical database can be established and further confirm that TRPML1 is a risk factor for lymphedema. Second, although mice are a major model organism for biological research, the mouse tail lymphedema model in this study could not completely imitate the complicated pathological environments of damaged lymphatic vessels in human patients. Thus, there are still considerable challenges to overcome in translating from experimental theory to clinical application. Finally, previous studies on AQP3 have focused more on the effects on skin physiology and renal water handling, and their biological function has already been clarified [47]. Further studies are needed to investigate the pathological mechanism of the role of AQP3, which is more dominantly expressed in HLECs, in influencing lymphedema. Besides, when we searched AQP3 at www.rcsb.org, we found that only the predicted structure of AQP3 is shown, and the actual crystal structure of AQP3 has not been revealed. Elucidating the crystal structure of AQP3 will help to further our understanding of the mode in which AQP3 mediates water molecule trafficking from a structural biology perspective. This will benefit studies on the molecular mechanisms underlying lymphedema as well as other diseases.

In this study, we identified the effect of TRPML1 on lymphedema, and the experimental results indicated that TRPML1 is likely to be an enabling factor for the development of lymphedema. We also investigated the TRPML1-mediated development of chronic inflammation in lymphedema. We explored the possible mechanism of lymphedema formation from the perspective of AQPs and lymphatic endothelial cell water permeability. The experimental results showed that TRPML1 affects the membrane localization of AQP3, -5 in direct correlation with the alteration of lymphatic endothelial cell water permeability, which is inevitably associated with the development of lymphedema. Confronted with the therapeutic dilemma of lymphedema, our results provide a novel idea for developing molecular therapies for lymphedema, and TRPML1 or aquaporins may become interventional targets for lymphedema. More advanced studies are expected to lead to the prospect of developing highly effective molecular drugs for the treatment of lymphedema.

## Conclusions

This study addresses a research gap concerning TRPML1 and AQP3, -5 in lymphedema inflammation. Our results provide primary evidence that TRPML1 regulates the cell membrane localization of AQP3, -5 in HLECs, resulting in enhanced permeability and the development of lymphedema, as accompanied by chronic inflammation. These findings provide insight for further lymphedema- and inflammation-related research, and the results may be helpful for guiding future analyses of lymphedema-associated inflammation.

## Supporting information

S1 Fig The characterization of TRPML1^−/−^ mice. To screen the genotypes of mice, we designed two pairs of primers. The primer sequence targeting outside the knockout fragment was F1 (TCTGAGCCATCTTACTGCCAACTG) and R1 (CCATGCTCTATTGATCAAAGCATCC). The primer sequence targeting inside the knockout fragment was F2 (CGGCAAACACGTTACCTACTGAGTC) and R2 (GGCCACTCTAAGATGCAAACAGTG). Then, we extracted mouse tail DNA for PCR reactions and performed agarose gel electrophoresis. *TRPML1* knockout mice had one positive PCR product for 242 bp. Heterozygous mice had two positive PCR products, including 242 bp and 415 bp. WT mice only had the 415 bp PCR product.
(TIF)

S1 Video The fluorescence change in cellular calcein in NC group under hypotonic stimulation. The fluorescence changes of calcein in HLECs under the 400× inverted fluorescence microscope were continuously recorded using the SharpCap 3.0 system with one image per second and composited into a 15-s video in MPEG format using Adobe Premiere Pro CC 2017 software.
(MP4)

S2 Video The fluorescence change in cellular calcein in ML-SA1 group under hypotonic stimulation. Only the fluorescence of HLECs after ML-SA1 stimulation showed a significant change, with the cell membranes generating a transient enhanced water flux, resulting in green-fluorescent bright spots that were different from the appearance of the other groups.
(MP4)

S3 Video The fluorescence change in cellular calcein in ML-SI1 group under hypotonic stimulation. (MP4)

S4 Video The fluorescence change in cellular calcein in ML-SA1+ML-SI1 group under hypotonic stimulation. (MP4)

S5 Video The fluorescence change in cellular calcein in BafA1 group under hypotonic stimulation. (MP4)

S1 Raw images (PDF)

S2 Raw images (PDF)

S1 Data (XLSX)
