# Supplementary figures and images for: TRPML1 acts as a predisposing factor in lymphedema development by regulating the subcellular localization of aquaporin-3, -5
Source: PLoS One. 2024 Dec 5;19(12):e0310653. doi: 10.1371/journal.pone.0310653 (PMC11620549; doi:10.1371/journal.pone.0310653)

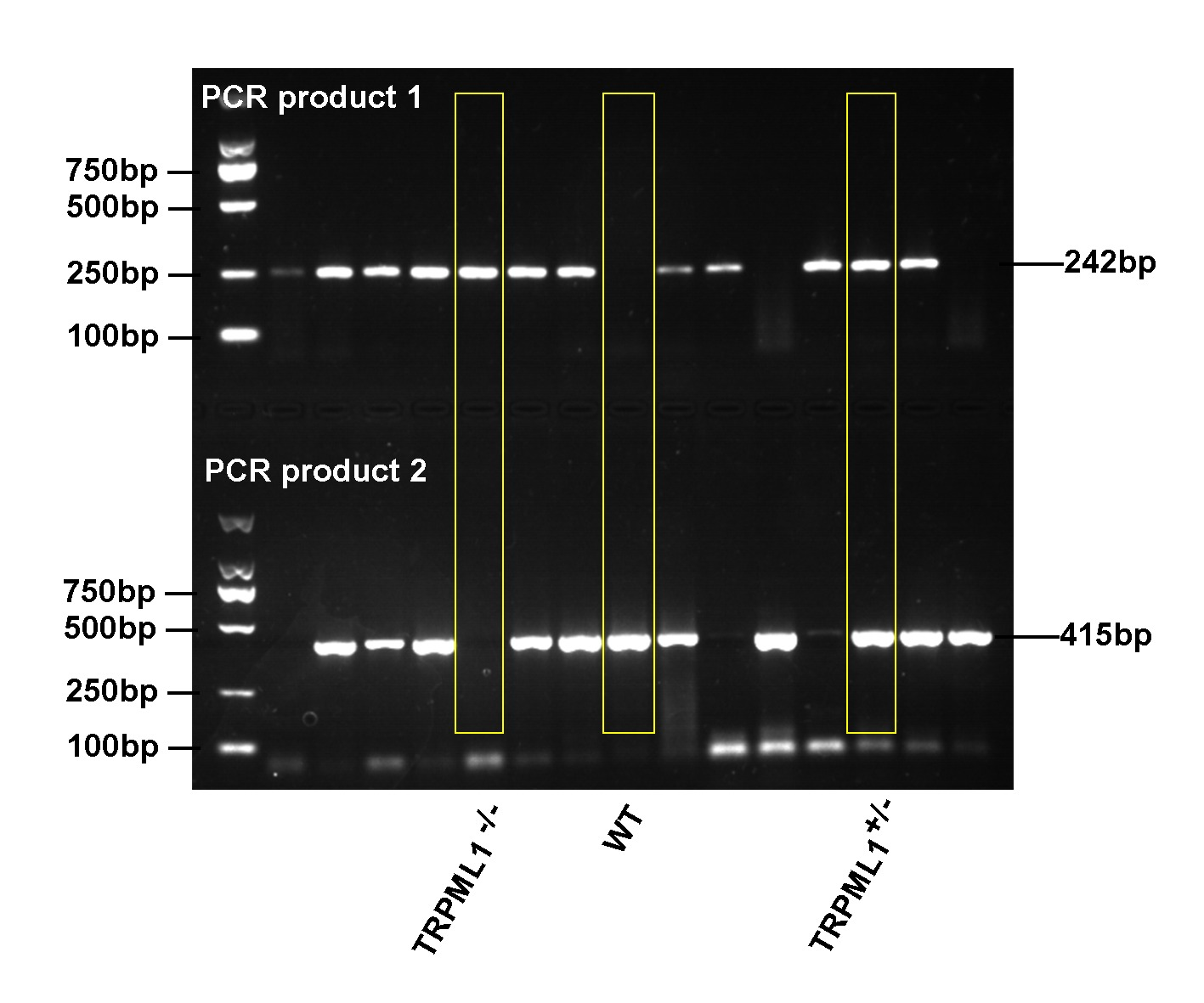

Supplement: S1 Fig — To screen the genotypes of mice, we designed two pairs of primers. The primer sequence targeting outside the knockout fragment was F1 (TCTGAGCCATCTTACTGCCAACTG) and R1 (CCATGCTCTATTGATCAAAGCATCC). The primer sequence targeting inside the knockout fragment was F2 (CGGCAAACACGTTACCTACTGAGTC) and R2 (GGCCACTCTAAGATGCAAACAGTG). Then, we extracted mouse tail DNA for PCR reactions and performed agarose gel electrophoresis. TRPML1 knockout mice had one positive PCR product for 242 bp. Heterozygous mice had two positive PCR products, including 242 bp and 415 bp. WT mice only had the 415 bp PCR product. (TIF) [file pone.0310653.s001.tif]

Fig 3C-AQP3

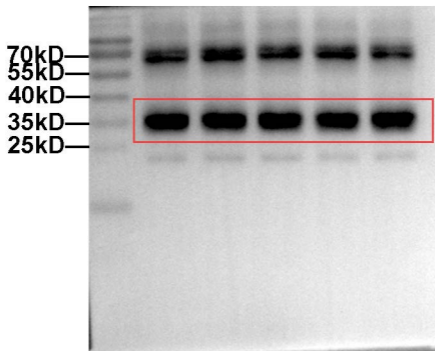

Fig 3C-AQP5

70kD—  
55kD—  
40kD—  
35kD—  
25kD—

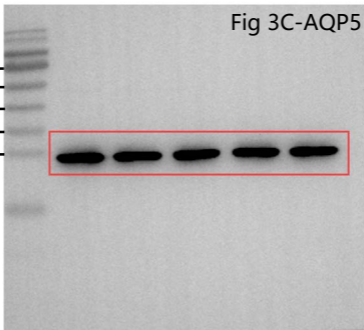

Fig 3C-GAPDH

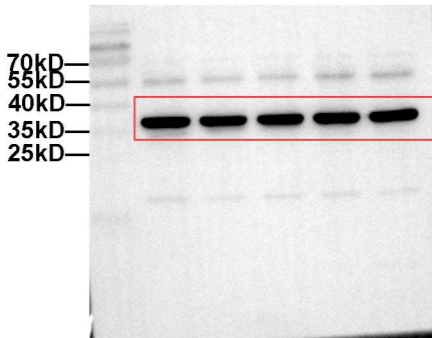

Supplement: S1 Raw images — (PDF) [file pone.0310653.s007.pdf]
