# Supplementary material for: TRPML1 acts as a predisposing factor in lymphedema development by regulating the subcellular localization of aquaporin-3, -5
Source: PLoS One. 2024 Dec 5;19(12):e0310653. doi: 10.1371/journal.pone.0310653 (PMC11620549; doi:10.1371/journal.pone.0310653)

Fig 4F-AQP3（cytosol)

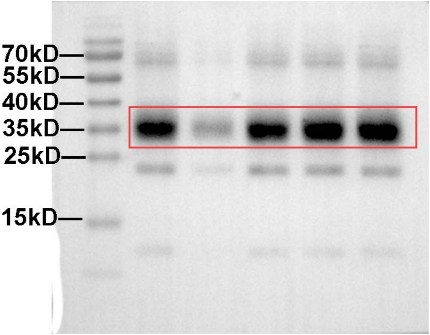

Fig 4F-AQP3(membrane)

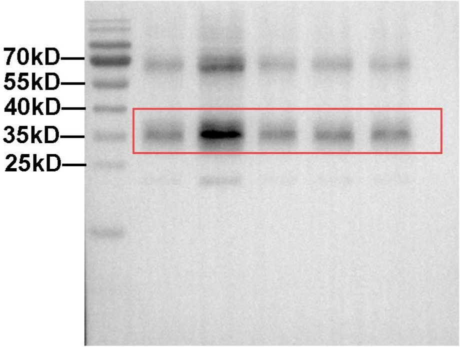

Fig 4F-GAPDH

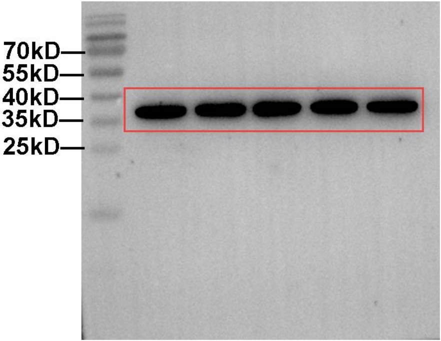

Fig 4F-Na+_K+ ATPase

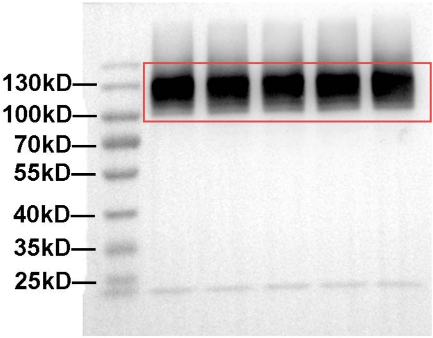

Fig 4G-AQP5 （cytosol）

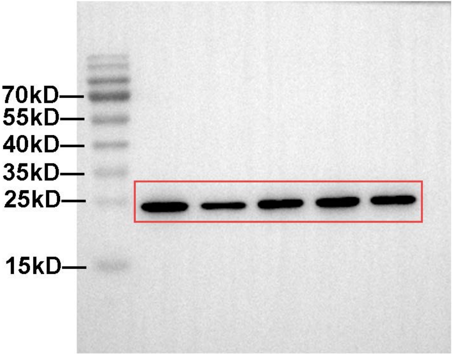

Fig 4G-AQP5(membrane)

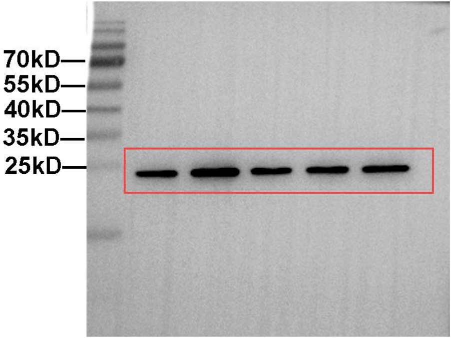

Fig 4G-GAPDH

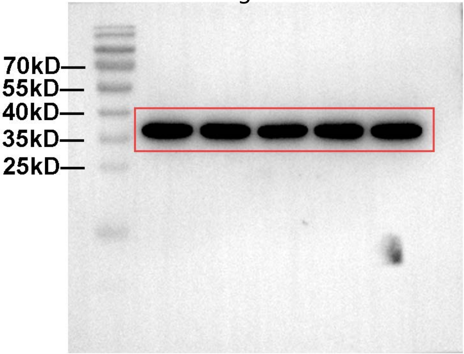

Fig 4G-Na+_K+ ATPase

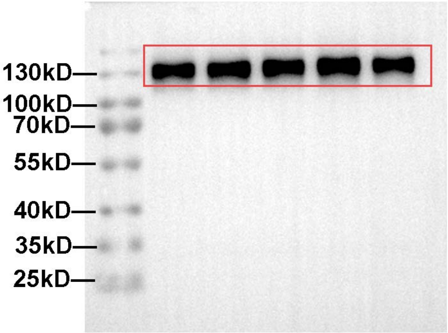

Supplement: S2 Raw images — (PDF) [file pone.0310653.s008.pdf]
